# Analysis of the Status and Tendency of R&D Input in the Field of Rare Diseases Funded by the National Natural Science Foundation of China

**DOI:** 10.3389/fpubh.2021.729162

**Published:** 2021-10-06

**Authors:** Hanyu Chang, Wei Chu, Xiaodan Li, Jing Ma, Dingguo Li, Ni Yuan

**Affiliations:** ^1^Department of Social Medicine, School of Public Health, Dalian Medical University, Dalian, China; ^2^Office of Medical Insurance, The First Hospital of Lanzhou University, Lanzhou, China; ^3^Xinhua Hospital Affiliated to Shanghai Jiao Tong University School of Medicine, Shanghai, China

**Keywords:** rare diseases, National Natural Science Foundation of China, R&D input, development tendency, funded

## Abstract

**Background:** Through collection and sorting of rare disease projects funded by the National Natural Science Foundation of China, an understanding was gained of the categories of projects funded by the foundation in the field of rare diseases, types of diseases, categories of disease systems, regional distribution, distribution of supporting institutions, and their dynamic changes, followed by an analysis of focuses and influences of relevant state policies. This will help improve the rare disease-relating policies of the state in supporting the key fields, thus promoting healthy and sustainable development in the field of rare diseases.

**Method:** Through the website of inquiry of projects funded by the National Natural Science Foundation of China, a retrieval was made concerning the projects funded by the foundation in the field of rare diseases during the period from 1986 to 2019, followed by descriptive analysis of fund input of rare disease projects, number of projects, temporal and regional distribution, and the analysis of the law of their dynamic changes.

**Result:** As of the end of 2019, there were 57 rare diseases and 678 related projects funded by the National Natural Science Foundation of China, with accumulated total funding of ¥ 253,525,000. Among the categories of projects, the most-funded projects were general (¥ 150,145,000, 59.22%), followed by Youth Foundation projects (¥ 53,719,000, 21.19%) and key projects (¥ 15,870,000, 6.26%); among the categories of disease systems, the most funded disease system was the nervous system (¥ 93,186,000, 37.76%), followed by the respiratory system (¥ 35,444,000, 13.98%); the most funded diseases were multiple sclerosis (¥ 34,870,000, 13.75%), idiopathic pulmonary fibrosis (¥ 29,854,000, 11.78%), and retinitis pigmentosa (¥ 27,005,000, 10.65%); the most funded regions were East China (¥ 106,987,000, 42.20%) and North China (¥ 71,844,000, 28.34%), while the least funded region was Northwest China (¥ 7,295,000, 2.88%); among the supporting institutions, the most funded institutions were Peking University (¥ 24,720,000, 9.75%), and Sun Yat-sen University (¥ 14,505,000, 5.72%).

**Conclusion:** With the promulgation of more policies on encouragement of innovation and accelerated approval procedures, etc., the National Natural Science Foundation of China has been increasing its funding to rare diseases, covering increasingly more categories of funded projects, more types of diseases, and wider regions. Nonetheless, the support for scientific research in China is still relatively weak. Therefore, it is proposed that the healthy and sustainable development in the course of rare diseases should be promoted through the improvement of relevant rare disease policies, encouragement of R&D of medicine for rare diseases, the establishment of special funds for rare diseases, acceleration of fund circulation, and combination of balanced development and preferential funding to key regions and major diseases.

## Background

A rare disease refers to a disease with a low prevalence and its definition may vary in different countries. The WHO defines rare diseases as one with prevalence between 0.65‰ and 1‰ (1, 2). In China, there is no official definition of rare diseases. Some experts believe that rare diseases may be defined as diseases with neonatal morbidity of 0.1‰ or prevalence of 0.002‰ (3). Due to the smaller number of patients with rare diseases, the prices of orphan drugs have been high. Some countries are making a new exploration by the establishment of special funds for the treatment of rare diseases, to reduce the burden of patients. For instance, the U.S. National Institute of Health has invested over US$ 10 billion in research of rare diseases in recent 10 years, significantly improving the initiatives in research of rare diseases and R&D of orphan drugs. In China, there is still a lack of legal systems relating to rare diseases and orphan drugs. In addition, the support for scientific research is considerably weak. In China, scientific research funds are divided into national funds (such as the National Natural Science Foundation of China (NSFC), National Scientific and Technology Support Fund, and the Fund for Key Projects of the Ministry of Science and Technology), provincial and ministerial funds (such as the natural science funds of the Ministry of Education, provincial science and technology departments, and other provincial natural science funds), and other funds. Nonetheless, the funds for research of rare diseases are mainly from the NSFC, which grants certain subsidies for research of rare diseases every year (4–6) and established the discipline of rare diseases in its Department of Health Sciences in 2021. In recent years, China has promulgated a series of policies to support the R&D of orphan drugs and constantly strengthened R&D input in the field of rare diseases. At the same time, the state is also paying higher attention to rare diseases. In the “Two Sessions” in 2021, National People's Congress and Chinese People's Political Consultative Conference members submitted the proposals on rare diseases, namely, enhancement of mechanism of prevention, treatment, and screening of rare diseases; establishment of a unified rare disease patients registration system; establishment of state-level rare disease diagnosis and treatment center; improvement of the green channel for launch orphan drugs; and multiparty sharing of cost in medical insurance system and “1 + N” multilayer safeguard (7).

The research on R&D investment in rare diseases in China is blank at present. Chen Xin and other scholars (8) found that there were 3,132 projects funded by NSFC from 2008 to 2013, totaling about 1.29 billion yuan. Zhang Yang and other scholars (9) found that the funding status of basic research projects on rare diseases at home and abroad is based on the “Dimension” database. There were 2,887 scientific research funding projects on rare diseases and their drugs, from 1976 to August 2019, involving 57 countries. Among them, 988 projects were funded by the United States, with a cumulative amount of US$ 2.1 billion, with the largest support. Since 2008, China has funded a total of 56 projects with a funding amount of $ 21 million. However, there is no secondary analysis of single diseases funded by NSFC based on the catalog due to the different definitions and catalog of rare diseases in various countries. The research of Meilin (10) shows that the existing research on rare diseases in China is mainly supported by NSFC, and the research funds related to rare diseases, the proportion of research funds in the overall health expenditure and the number of research carried out have increased. However, the aforementioned studies have raised common problems that there is no clear definition of rare diseases, less R&D investment, and lack of a special funding system.

In 1986, the NSFC was established (11). At present, receipt of funds from NSFC has been an important indicator for measuring whether a unit has the qualification and capacity for high-level research and for assessing its academic research and position. The foundation has attracted a majority of scholars who make significant contributions to Chinese science and technology, providing support to and guidance on domestic innovation in key researches. In this paper, an analysis is made focusing on R&D input in the field of rare diseases by NSFC during the period from January 1, 1986, to December 31, 2019, and it analyzes the status and tendency of R&D input in the field of rare diseases funded by NSFC. The overall R&D inputs, types of projects, categories of disease systems, types of diseases, geographical regions, and supporting institutions of rare diseases by NSFC, and also their dynamic changes, followed by an analysis of focuses and influences of relevant state policies, thus providing references for optimization of funding orientation in the field and prevention and treatment of rare diseases.

## Method

Based on the 121 types of rare diseases in the *Catalog of the First Batch of Rare Diseases* issued by the National Health Commission in May 2018, a retrieval was made by the type of diseases on the website of “inquiry of projects funded by the National Natural Science Foundation of China” (http://fund.zsci.com.cn/Index/index.html) for the period from January 1, 1986, to December 31, 2019. Then a descriptive analysis was made on the quantity and category of funded projects, category of disease systems, type of diseases, regional distributions, and supporting institutions, thus summarizing the characteristics and tendency of such funding.

## Result

### The Overall R&D Input in the Field of Rare Diseases by NSFC

**Figure 1:**
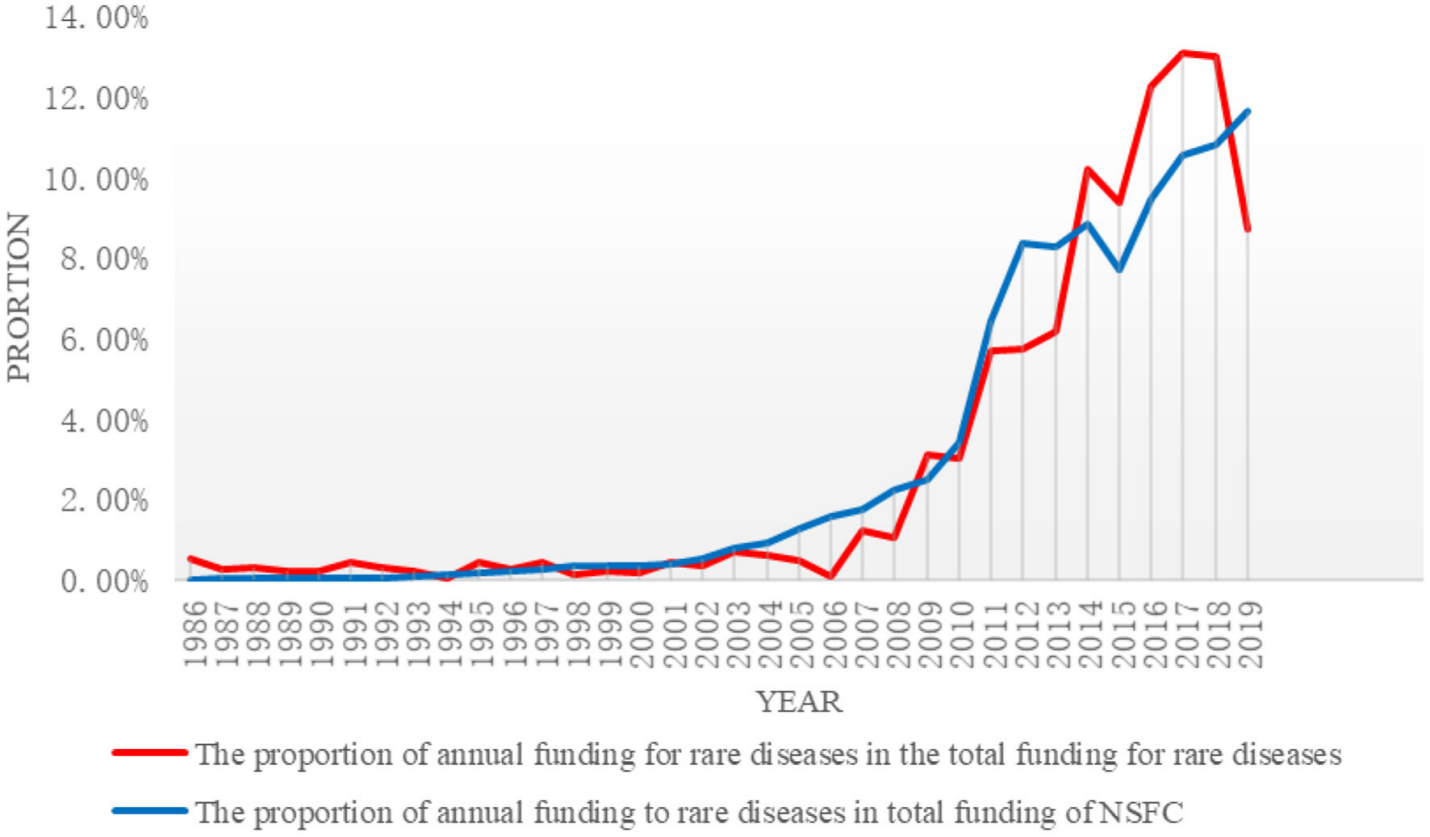
The proportion of funding to rare diseases in the total funding of NSFC and the changes.

As of the end of 2019, a total of 658 projects were founded by NSFC, with accumulated total funding of ¥ 253,525,000. The supported projects were increased from two projects in 1986 to 54 projects in 2019, an increase of 2,600%; the total funding was increased from ¥ 1,350,000 in 1986 to ¥ 22,090,000 in 2019, an increase of about 15.4 times. Please refer to Figure 1 for details. The proportion of annual funding to rare diseases in the total annual funding of NSFC was relatively stable (<1% in all years). With increased total funding, the funding to rare diseases was increased accordingly, suggesting a gradual growth.

The proportion of annual funding to rare diseases in total funding of NSFC was almost unchanged. In this study, the annual funding to rare diseases by NSFC was divided into three stages by their proportion (<1, 1–5, and >5%) in the total funding: the initial stage (1986–2006); the slow growth stage (2007–2010); and the fast growth stage (2011–2019). Please refer to Table 1 for details.

**Table 1:** R&D input in rare diseases by NSFC and the changes.

**Year**	**Total projects (each)**	**Total funding (00,000 yuan)**	**Average funded projects (projects/year)**	**Average annual fund (x ± s)**	**Maximum**	**Minimum**	**Accumulated proportion (%)**
1986–2006	71	1,818.50	3.38	86.604 ± 1.82	182.50	18.50	7.17
2007–2010	79	2,129.70	19.75	532.432 ± 44.72	787.50	265.70	8.40
2011–2019	508	21,404.30	56.44	2,378.267 ± 25.14	3,327.00	1,447.00	84.43
Total	658	25,352.50	19.35	745.661 ± 061.36	3,327.00	18.50	100.00

At the initial stage, there were the fewest projects funded by NSFC and the least amount of funding. The proportion of annual funding to rare diseases in the total funding of NSFC was relatively low, with a smooth trend and low growth rate. At the slow growth stage, there was a minor change in funding to rare diseases. The funding was increased from ¥ 3,110,000 in 2007 to ¥ 7,655,000 in 2010. During the 4 years, the total funding was ¥ 2,1297,000, which is two times the funding at the initial stage. On average, 19.75 projects were funded every year and the amount of funding was ¥ 5,324,300. During this period, the funding was the highest in 2009 (¥ 78,750,000) and the lowest in 2007 (¥ 3,110,000). The proportion of funding increased to 3.02% and the accumulative proportion increased by 8.40%. Although there is not much difference compared with the accumulated proportion at the initial stage, it can be seen that the increase was significant at the slow growth stage. At the fast growth stage, on average, 56 projects were funded every year and the amount of funding was ¥ 23,782,600. The highest funding was ¥ 33,270,000 in 2017 and the lowest funding was ¥ 14,470,000 in 2011, suggesting a significant growth on the whole. It can be seen that funding to the field of rare diseases in recent years has been enormous: the annual funding exceeded ¥ 30,000,000 in the 3 years from 2016 to 2018 and the accumulated proportion in the 9 years was 84.43%.

### R&D Input in Rare Diseases Among the Projects Funded by NSFC

Among the projects funded by NSFC, the most funded projects were general projects (¥ 150,145,000) which accounted for 59.22% of the total funding. This suggested that over half of rare disease projects funded by NSFC were general projects, followed by Youth Science Foundation projects (¥ 53,719,000) which accounted for 21.19% of the total funding or, namely, about one-quarter; although the total funded key projects were relatively fewer, its total funding ranked the third (¥ 15,870,000), with an average funding per project of ¥ 2,645,000. Please refer to Table 2 for details. At all three stages, there were funded general projects, Youth Science Foundation projects, and regional science foundation projects, with a sharp growth trend. At the initial stage and the slow growth stage, the funded key projects, major research plans, and other projects were relatively fewer and their funding was relatively greater at the fast growth stage.

**Table 2:** R&D input in rare diseases among the projects funded by NSFC.

**Categories of projects**	**Total projects (each)**	**Total funding (00,000 yuan)**	**Average funding per project (00,000/project)**	**Proportion (%)**
General projects	309	15,014.50	48.59	59.22
Youth Foundation projects	267	5,371.90	20.12	21.19
Key projects	6	1,587.00	264.50	6.26
Regional science foundation projects	33	1,229.70	37.26	4.85
International (regional) cooperation and exchange projects	11	1,130.40	102.76	4.46
Major research plans	4	450.00	112.50	1.77
Outstanding Youth Foundation projects	2	220.00	110.00	0.87
Emergency management projects	11	185.00	16.82	0.73
Special fund projects	13	126.00	9.69	0.50
Cooperative research fund for overseas, Hong Kong and Macau scholars	2	38.00	19.00	0.15
Total	658	25,352.50	38.53	100.00

### R&D Input in Rare Diseases by NSFC by the Category of Disease Systems

Concerning the categories of rare disease systems, the top three disease systems with the highest funding were the nervous system (¥ 93,186,000, 36.76%), the respiratory system (¥ 35,444,000, 13.98%), and the blood and hematopoietic system (¥ 23,525,000, 9.28%). The average funding per project was the highest for the cardiovascular system, which accounted for 71.04% and the lowest for malignant tumors, which accounted for 27.64%. Please refer to Table 3. At the initial stage, the blood and hematopoietic system received the most funding. At the slow growth stage and the fast growth stage, the funding to blood and the hematopoietic system was increased significantly, with its total funding ranked the first. At the fast growth stage, the increase of funding to the respiratory system was second only to that of funding to the nervous system.

**Table 3:** R&D input in rare diseases by NSFC by the categories of disease systems.

**Ranking**	**Category of disease systems (12)**	**Total projects (each)**	**Total funding (00,000 yuan)**	**Average funding per project (00,000 yuan)**	**Proportion (%)**
1	Nervous system	244	9,318.60	38.19	36.76
2	Respiratory system	79	3,544.40	44.87	13.98
3	Blood and hematopoietic system	72	2,352.50	32.67	9.28
4	Cardiovascular system	28	1,989.00	71.04	7.85
5	Metabolic system	46	1,457.00	31.67	5.75
6	Malignant tumors	41	1,133.50	27.64	4.47
7	Muscles and bones	16	535.50	33.47	2.11
8	Endocrine system	5	261.00	52.20	1.03
9	Urogenital system and gonadal hormone	8	252.00	31.50	1.00
10	Digestive system	4	147.00	36.75	0.58
	Other	115	4,362.00	37.93	17.19
	Total	658	25,352.50	38.53	100.00

### R&D Input in Rare Diseases by NSFC by the Type of Diseases

**Figure 2:**
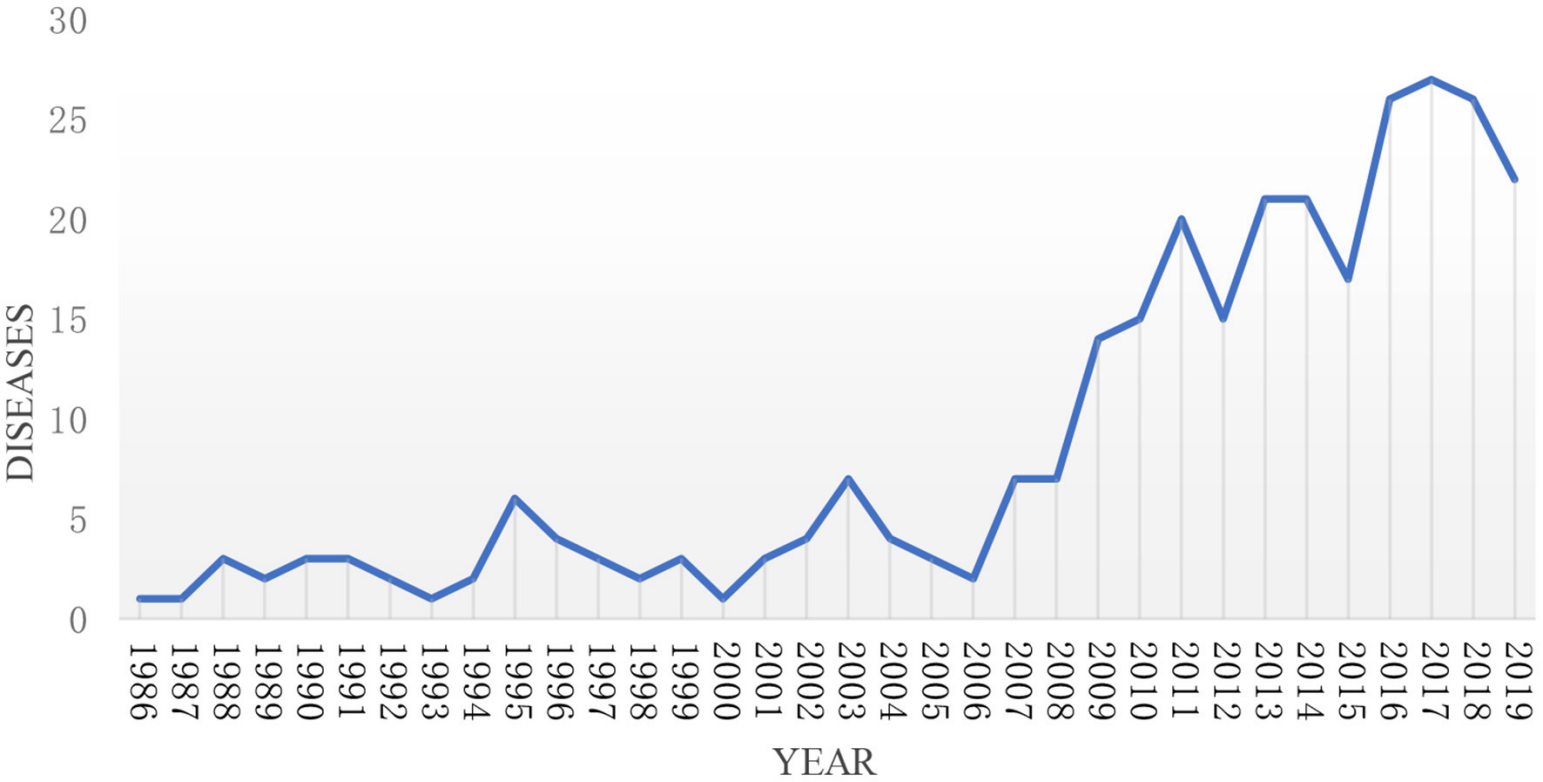
The trend of the types of rare diseases funded by NSFC and its change every year from 1986 to 2019.

**Figure 3:**
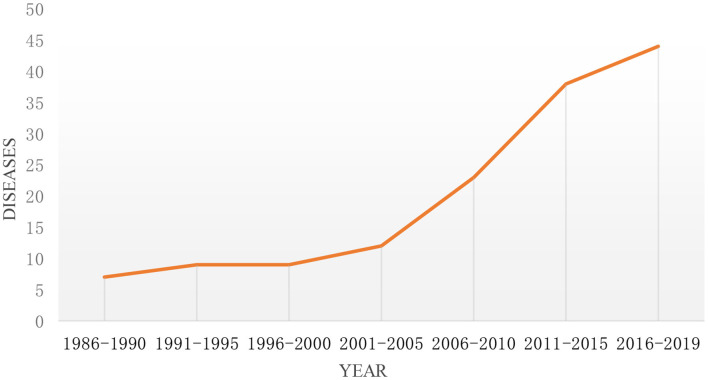
The trend of the types of rare diseases funded by NSFC and its change every 5 years from 1986 to 2019.

As of the end of 2019, there were 57 rare diseases funded by the NSFC. As can be seen from Figure 2, the types of rare diseases funded by NSFC in recent 20 years from 1986 to 2008 are <10. It suggests that the types of funds are limited and fixed. From 2008 to 2019, the number of rare diseases projects funded by NSFC varied from 14 to 27, which deserves mentions that it reached a maximum in 2017. For the sake of better describing the figure change over time clearly, the study divided every 5 years into one period for further analysis, and the changing trend is shown in Figure 3. It can be seen from the figure that the number of rare diseases funded by NSFC fluctuated between 5 and 15 every 5 years, and the fluctuation range was very small from 1986 to 2005. The types of rare diseases funded increased rapidly every 5 years after 2005.

The most funded rare disease by NSFC was multiple sclerosis (¥ 34,870,000, 13.75%), followed by idiopathic pulmonary fibrosis (¥ 29,854,000, 11.78%) and retinal pigment degeneration (¥ 27,005,000, 10.65%). The rare disease with the least funded projects and funding was systemic sclerosis (¥ 5,110,000, 2.02%). The average funding per project was the highest for homocystinemia and the amount was ¥ 717,600, indicating NSFC gave strong support to it; the average funding per project was the lowest for retinal pigment degeneration and the amount was ¥ 276,500. The maximum funding was about two times the minimum funding, indicating there was a priority in the provision of funding to diseases. Please refer to Table 4. At the initial stage, NSFC provided relatively more funding to multiple sclerosis, retinal pigment degeneration, Wilson disease, retinoblastoma, and hemophilia. At the following two stages, the funding to foregoing diseases was also gradually increased, accounting for a large proportion on the whole. Among the other diseases, nearly over half of them were not funded at the initial stage and the slow growth stage. At the fast growth stage, the funded types of diseases were the most with the highest funding.

**Table 4:** R&D input in the main types of rare diseases by NSFC.

**Ranking**	**Type of diseases**	**Total projects (each)**	**Total funding (00,000 yuan)**	**Average funding per project (00,000 yuan)**	**Proportion (%)**

1	Multiple sclerosis	96	3,487.00	36.32	13.75
2	Idiopathic pulmonary fibrosis	71	2,985.40	42.05	11.78
3	Retinal pigment degeneration	72	2,700.50	37.51	10.65
4	Amyotrophic lateral sclerosis	51	2,312.10	45.34	9.12
5	Homocystinemia	25	1,794.00	71.76	7.08
6	Hemophilia	55	1,750.50	31.83	6.90
7	Neuromyelitis optica	29	1,194.00	41.17	4.71
8	Retinoblastoma	41	1,133.50	27.65	4.47
9	Hereditary spastic paraplegia	16	532.00	33.25	2.10
10	Charcot-Marie-Tooth disease	17	512.50	30.15	2.02
11	Systemic sclerosis	14	511.00	36.50	2.02
	Other	171	6,440.00	37.66	25.40
	Total	658	25,352.50	38.53	100.00

### R&D Input in Rare Diseases by NSFC by Seven Major Geographical Regions

The most funded regions by NSFC were East China (¥ 106,987,000) and North China (¥ 718,440,000), while the region least funded by NSFC was Northwest China (¥ 7,295,000). In East China, the most funded municipality was Shanghai, and the funding was ¥ 52,325,000 which was significantly higher than that in other provinces or municipalities; in North China, the most funded municipality was Beijing, and the funding was ¥ 624,840,000 which was significantly higher than that in Shanghai. However, other provinces and municipalities in this region received relatively less funding, suggesting the uneven distribution of funding. Please refer to Table 5 for details. At the initial stage, Shanghai, Beijing, Sichuan, and Anhui received significantly higher funding than other areas. However, Guizhou, Heilongjiang, Shandong, and Shaanxi did not receive any funding until the slow growth stage. At all three periods, Beijing and Shanghai, etc. always received higher funding than other areas.

**Table 5:** R&D input in rare diseases by NSFC by seven major geographical regions.

**Administrative regions**	**Total projects (each)**	**Total funding (00,000 yuan)**	**Average funding per project (00,000 yuan)**	**Maximum**	**Minimum**
Northeast China	37	1,692.00	45.73	877.00	322.50
North China	180	7,184.40	39.91	6,248.40	35.00
East China	270	10,698.70	39.62	5,232.50	334.00
Central China	68	2,231.50	32.82	1,058.50	442.00
South China	27	1,203.00	44.56	999.00	50.00
Northwest China	21	729.50	34.74	317.50	89.00
Southwest China	55	1,613.40	29.33	891.40	79.00
Total	658	25,352.50	38.53	6,248.40	35.00

### Supporting Institutions Funded by NSFC Concerning Rare Diseases

It can be learned from Table 6 that, the top three supporting institutions that received the most funding were Peking University (¥ 24,720,000, 9.75%), Sun Yat-Sen University (¥ 14,505,000, 5.72%), and Shanghai Jiao Tong University (¥ 11,550,000, 4.56%), which was consistent with the funding received by the seven geographical regions. Among the top 10 supporting institutions, Tongji University ranked fourth concerning the amount of funding which reached ¥ 11,390,000, although its funded projects were the least. The average funding per project was ¥ 876,200/project, being the highest among all other supporting institutions. At the initial stage, a total of 34 supporting institutions received funding. Among them, the Sun Yat-Sen University, the Sichuan University, and the Anhui University of Chinese Medicine received more funding, which was consistent with the funding received by foregoing regions; at the slow growth stage, nearly one-third of supporting institutions received funding; at the fast growth stage, nearly all supporting institutions received funding. No matter at the slow growth stage or the fast growth stage, Peking University always ranked first concerning the amount of funding.

**Table 6:** Supporting institutions funded by NSFC concerning rare diseases.

**Ranking**	**Supporting institutions**	**Total projects (each)**	**Total funding (00,000 yuan)**	**Average funding per project (00,000 yuan)**	**Proportion (%)**
1	Peking University	46	2,472.00	53.74	9.75
2	Sun Yat-Sen University	45	1,450.50	32.23	5.72
3	Shanghai Jiao Tong University	30	1,155.00	38.50	4.56
4	Tongji University	13	1,139.00	87.62	4.49
5	Capital Medical University	30	1,071.50	35.72	4.23
6	Central South University	33	1,021.5	30.95	4.03
7	Harbin Medical University	15	877.00	58.47	3.46
8	Fudan University	27	794.50	29.43	3.13
9	Peking Union Medical College Hospital	19	760.00	40.00	3.00
10	Zhejiang University	18	703.00	39.06	2.77
	Other	382	13,908.5	36.70	54.86
	Total	658	25,352.5	38.53	100.00

## Discussion

On the whole, the support of NSFC to the field of rare diseases has witnessed gradual growth in terms of both the number of projects and the amount of funding. The input has been constantly increased. In recent years, in particular, the amount of funding has been increased significantly, which is closely contributable to the high attention to rare diseases paid by the state in recent years, the establishment of the Rare Diseases Society and Local Rare Diseases Society of Chinese Research Hospital Association and China Alliance of Rare Diseases, the construction of National Rare Diseases Registry System of China, and the work on clinical cohort study (9). In October 2017, the State Council issued the *Opinions on Deepening the Reform of the Examination and Approval System to Encourage the Innovation of Drugs and Medical Devices*, under which support to R&D of orphan drugs and medical devices was stressed; in January 2018, the former China Food and Drug Administration and the Ministry of Science and Technology issued the *Guidance to Strengthening and Promotion of Scientific and Technological Innovation in Food and Drugs*, under which prior support to R&D of drugs and medical devices for treatment of rare diseases was also proposed; in March 2018, the State Council issued the *Opinions on Reform and Improvement of Supply Guarantee for Generic Drugs and the Usage Policies*, under which development of generic drugs for treatment and prevention of major infectious diseases and for treatment of rare diseases was encouraged; in May 2018, the National Health Commission published the *Catalog of the First Batch of Rare Diseases*; in October 2019, the State Council issued the *Opinions on Promoting Inheritance, Innovation and Development of Traditional Chinese Medicine*, under which clinical researches of rare diseases were proposed. All these policies pointed out the R&D of orphan drugs, thus promoting the development of orphan drugs in China (13, 14).

As the most important part of the NSFC, the general projects were mostly funded in terms of both the number of funded projects and the amount of funding, accounting for the greatest portion in R&D input in rare diseases with over 50%. This indicates that general projects are the key field funded by NSFC. As to other projects, the number of funded projects and the amount of funding varied due to their different establishment time and application conditions.

East China and North China were mostly funded by NSFC. In these two regions, the funding received by Shanghai and Beijing was most outstanding. This is because first-tier cities possessed richer resources and more high-level talents, providing favorable conditions for experts to apply for funding from NSFC. However, the funding received by different regions varied due to limited research capacity. Taiwan, Hong Kong Special Administrative Region, Macau Special Administrative Region, Tibet Autonomous Region, and Qinghai Province had not received funding. In the future, R&D input in the foregoing five provinces should be increased.

National Natural Science Foundation of China makes the greatest R&D input in rare diseases relating to the nervous system. Among them, multiple sclerosis, amyotrophic lateral sclerosis, neuromyelitis optica, hereditary spastic paraplegia, and Charcot-Marie-Tooth disease accounted for nearly half of the top 10 diseases. Although the rare diseases relating to the nervous system did not rank top three at the initial stage concerning received funding, they were always mostly funded at the slow growth stage and the fast growth stage, indicating that the state gradually pays more attention to the diseases relating to the nervous system. In this category of diseases, multiple sclerosis was mostly funded. Multiple sclerosis is an autoimmune disease with the central nervous system which often occurred in young and middle-aged people. It was mostly funded by NSFC, probably because its incidence in China was higher than that in European and American countries and the drug price was also higher (15).

## Conclusions

To sum up, currently, diagnosis and treatment of rare diseases and R&D of orphan drugs are still at the initial stage. The orphan drugs cannot satisfy the demand of patients due to high prices and low availability. Although NSFC has increased R&D input in rare diseases with the increased funds, the growth is insignificant and relatively smooth on the whole, accounting for about 0.02%. Therefore, the input in rare diseases should be still increased. The rights and interests of rare disease patients may be safeguarded concretely through the encouragement of innovation in R&D of orphan drugs by experts and scholars, optimization of approval procedures, the inclusion of rare disease into medical insurance, and preparation and improvement of relevant policies (16). Therefore, it is required that the special fund should be gradually increased for promulgation and improvement of policies relating to orphan drugs; that the definition of rare diseases in China should be facilitated; and that more funds should be provided to research teams for R&D of orphan drugs. At the same time, higher attentions should be paid to basic research of rare diseases; R&D input in rare diseases with lower prevalence should be increased; and the preparation of standards for diagnosis and rare diseases should be accelerated. In addition, the input in rare diseases in different administrative regions should be balanced, so that more funds may be used in regions and types of diseases in most urgent need. Finally, while balancing the input in other types of diseases, the priority of funding should be given to R&D of orphan drugs for diseases that are hardly curable with high treatment costs and greatly affect the quality of life of patients, thus, promoting fast and sustainable development in the treatment of rare diseases.

## Data Availability Statement

Publicly available datasets were analyzed in this study. This data can be found here: http://fund.zsci.com.cn/Index/index.html.

## Author Contributions

HC was a major contributor in writing and reviewing the manuscript. WC contributed to methodology, statistics, and charting. XL and JM contributed to data collection. NY and DL contributed to the conception and design of the study. All the authors read and approved the submitted version.

## Funding

This research is supported by Shanghai Civil Affairs Bureau (Grant Number: SQ19-1214).

## Conflict of Interest

The authors declare that the research was conducted in the absence of any commercial or financial relationships that could be construed as a potential conflict of interest.

## Publisher's Note

All claims expressed in this article are solely those of the authors and do not necessarily represent those of their affiliated organizations, or those of the publisher, the editors and the reviewers. Any product that may be evaluated in this article, or claim that may be made by its manufacturer, is not guaranteed or endorsed by the publisher.
